# Cell-mimicking polyethylene glycol-diacrylate based nanolipogel for encapsulation and delivery of hydrophilic biomolecule

**DOI:** 10.3389/fbioe.2023.1113236

**Published:** 2023-01-17

**Authors:** Wen Jie Melvin Liew, Yee Shan Wong, Atul N. Parikh, Subbu S. Venkatraman, Ye Cao, Bertrand Czarny

**Affiliations:** ^1^ School of Materials Science and Engineering, Nanyang Technological University, Singapore, Singapore; ^2^ Biomedical Engineering, School of Engineering, Temasek Polytechnic, Singapore, Singapore; ^3^ Biomedical Engineering and Materials Science and Engineering, University of California, Davis, Davis, CA, United States; ^4^ School of Materials Science and Engineering, National University of Singapore, Singapore, Singapore; ^5^ Institute of Blood Transfusion, Chinese Academy of Medical Sciences and Peking Union Medical College, Chengdu, China; ^6^ Lee Kong Chian School of Medicine, Nanyang Technological University, Singapore, Singapore

**Keywords:** cell-mimicking, nanolipogel, encapsulation, delivery, hydrophilic biomolecules

## Abstract

Lipid based nanoparticulate formulations have been widely used for the encapsulation and sustain release of hydrophilic drugs, but they still face challenges such as high initial burst release. Nanolipogel (NLG) emerges as a potential system to encapsulate and deliver hydrophilic drug while suppressing its initial burst release. However, there is a lack of characterization of the drug release mechanism from NLGs. In this work, we present a study on the release mechanism of hydrophilic Dextran-Fluorescein Isothiocyanate (DFITC) from Poly (ethylene glycol) Diacrylate (PEGDA) NLGs by using different molecular weights of PEGDA to vary the mesh size of the nanogel core, drawing inspiration from the macromolecular crowding effect in cells, which can be viewed as a mesh network of undefined sizes. The effect is then further characterized and validated by studying the diffusion of DFITC within the nanogel core using Fluorescence Recovery after Photobleaching (FRAP), on our newly developed cell derived microlipogels (MLG). This is in contrast to conventional FRAP works on cells or bulk hydrogels, which is limited in our application. Our work showed that the mesh size of the NLGs can be controlled by using different Mw of PEGDA, such as using a smaller MW to achieve higher crosslinking density, which will lead to having smaller mesh size for the crosslinked nanogel, and the release of hydrophilic DFITC can be sustained while suppressing the initial burst release, up to 10-fold more for crosslinked PEGDA 575 NLGs. This is further validated by FRAP which showed that the diffusion of DFITC is hindered by the decreasing mesh sizes in the NLGs, as a result of lower mobile fractions. These findings will be useful for guiding the design of PEGDA NLGs to have different degree of suppression of the initial burst release as well as the cumulative release, for a wide array of applications. This can also be extended to other different types of nanogel cores and other nanogel core-based nanoparticles for encapsulation and release of hydrophilic biomolecules.

## 1 Introduction

The majority of nanoparticle-based formulations for delivery of hydrophilic drugs and biomolecules exhibits a “burst” release of the encapsulated contents upon administration in a couple of hours (Huang and Brazel, 2001). In most drug delivery applications, burst release is undesirable due to the shorter duration of action, which leads to higher dosage frequency to achieve the therapeutic effect; in addition, and more importantly, the burst may lead to local and systemic toxicity (Huang and Brazel, 2001). For chronic conditions, higher dosage frequency will result in a huge burden from the higher treatment cost and may result in liver toxicity (Sequeira et al., 2019). Thus, there is a need for better systems to control the burst release of hydrophilic therapeutics, and to achieve the desired sustained effect. Lipid based nanoparticles, such as liposomes, have been evaluated for the delivery of hydrophilic cargos (Wang and Wang, 2013). Liposomes give a modifiable alternative to other delivery systems, as its properties can be altered to suit various needs, such as improved systemic circulation, targeted delivery and biocompatibility, by using method such as PEGylation (Blume et al., 1993). Liposomes can also hold both hydrophobic molecules within the bilayer, as well as hydrophilic molecules in the aqueous core (Torchilin, 2005). However, liposomes still suffer from several shortcomings, which include the burst release of hydrophilic cargo (Samad et al., 2007; Kraft et al., 2014; Ye and Venkatraman, 2019) and the immunogenicity issues associated with repeated administration. (Wang et al., 2007; Schellekens et al., 2013; Zhang et al., 2016). Therefore, there is a lot of research activity related to the suppression of the burst release.

One of those approaches taken was using nanolipogel (NLG), in which the hydrophilic small-molecule drugs or biomolecules are encapsulated within the gelated core of the NLG, surrounded by a lipid bilayer (Ramanathan et al., 2016; Guo et al., 2018; Cao et al., 2020). Current studies have demonstrated that NLGs has successfully been used to encapsulate and suppress the burst release of hydrophilic drugs and biomolecules, such as doxorubicin hydrochloride (Yu et al., 2018), Dextran-Fluorescein Isothiocyanate (DFITC) (Anselmo et al., 2015), maraviroc and tenofovir disoproxil fumarate (Ramanathan et al., 2016). However, the release mechanisms were not studied in detail and the diffusion characteristics were not characterized. In those examples, the initial burst release was shown to be suppressed to various degrees but the release was monitored for only 8 h (Yu et al., 2018) or only up to 3 days (Ramanathan et al., 2016). There is also a lack of characterization of the diffusion of encapsulated drug within the nanogel core of the NLGs, which gives limited insight into the design of NLG delivery system.

**FIGURE 1:**
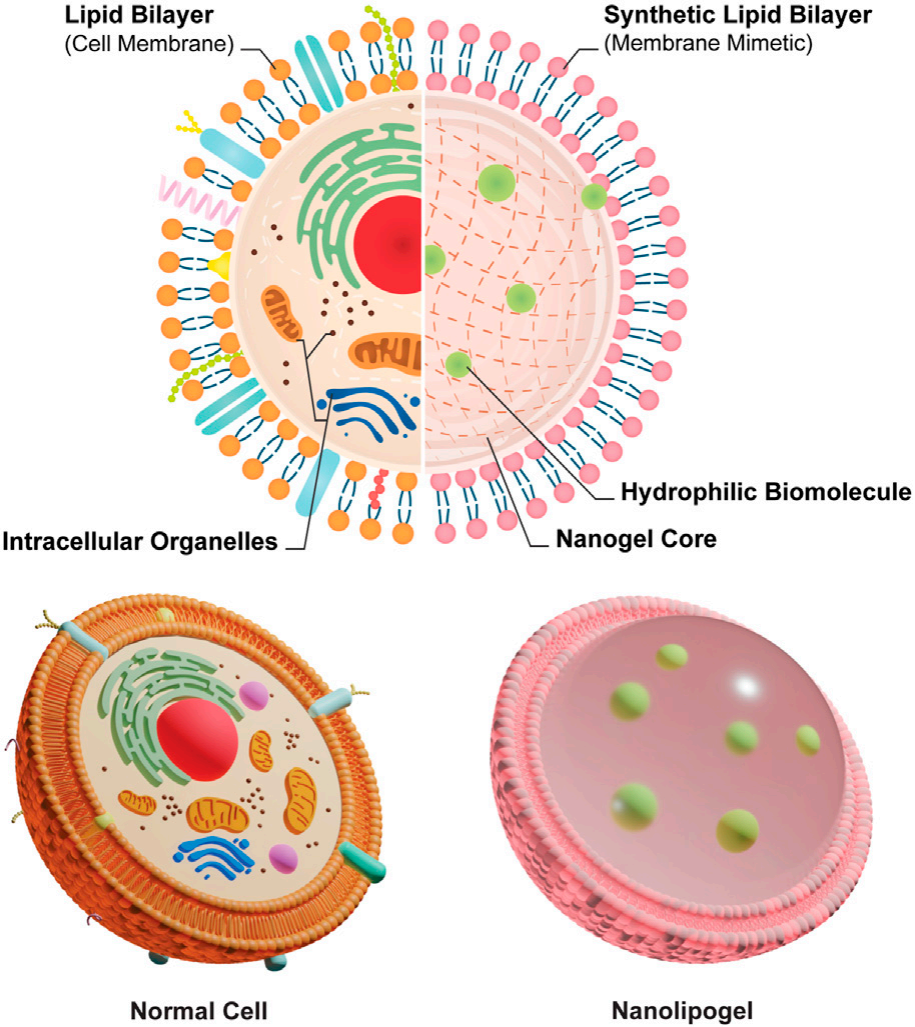
Schematics of the comparison between normal cell and cell mimetic nanolipogels.

Therefore, we adopted the design of PEGDA NLG (Cao et al., 2020) for a mechanistic study on the release of encapsulated biomolecule, where inspiration was drawn from the concept of macromolecular crowding phenomenon in cells (Ellis, 2001; Zhou et al., 2008; Soleimaninejad et al., 2017). It is known that the intracellular diffusion of proteins, or other biomolecules, in the cytoplasm can be hindered by a phenomenon loosely termed as “macromolecular crowding”, where intracellular organelles and macromolecules exhibit a crowding effect to hinders the diffusion of intracellular proteins. Building upon this concept, the NLG system possesses a nanogel core surrounded by a lipid bilayer, in which these structure mimics the crowding effect in cells, hindering the release of hydrophilic biomolecules, as depicted in Figure 1. The encapsulated biomolecules will be held within the mesh network of the nanogel core, in which it could be tailored to give different crosslinking densities and mesh sizes, restricting the diffusion of the encapsulated biomolecules (Cao et al., 2020). From this, we are able to vary the mesh size and crosslinking density of the PEGDA NLG by using different MW of PEGDA, for a mechanistic study on the diffusion and release of encapsulated biomolecule. Some of the commonly used method for diffusion studies include Fluorescence Recovery After Photobleaching (FRAP), Single Particle Tracking (SPT) and Fluorescence Correlation Spectroscopy (FCS), with FRAP being the most used method (Mika and Poolman, 2011; Schavemaker et al., 2018). However, SPT is limited by its high sensitivity to background fluorescence and FCS is more suited for giant-unilamellar vesicles (GUV), which isn’t suited for our application. On the other hand, FRAP has attained higher level of success in studying macromolecular mobility, especially in cells (Mika and Poolman, 2011), which can be fitted to our diffusion study by developing cell derived microlipogels (MLG). Hence, we have chosen to perform FRAP as our method for a mechanistic study into the diffusion and release of encapsulated biomolecule in PEGDA NLG. For that, we have newly developed the method of using cell derived MLGs to perform FRAP, to overcome the difficulty in performing FRAP on NLGs. This is also a novel approach, as compared to conventional methods of FRAP that are done on cells (Mika and Poolman, 2011; Schavemaker et al., 2018) or bulk hydrogel (Cha et al., 2011).

In this study, we fabricated NLGs with PEGDA of three different molecular weights (575 Da, 2000 Da and 4,000 Da) to mimic the cellular macromolecular crowding effect and systemically investigate the diffusion mechanism of hydrophilic cargos within the nanogel core in NLGs with varied extent of macromolecular crowding through different crosslinking densities. PEGDA is known to be biocompatible, non-toxic, immunologically inert and has been used in various biomolecule delivery studies (Sabnis et al., 2009; Durst et al., 2011; Liu et al., 2017; Stillman et al., 2020). Also, the PEG moiety is degradable *in vitro* and *in vivo* without toxic by-products (Browning et al., 2014; Stillman et al., 2020). Dextran-Fluorescein Isothiocyanate (DFITC) is used as a hydrophilic molecule for the release studies. Fluorescence Recovery after Photobleaching (FRAP) have been done to study the diffusion of encapsulated molecules and the mechanism for release, using cell derived MLGs that we have developed.

## 2 Materials and methods

### 2.1 Materials

L-α-phosphatidylcholine (Egg, Chicken) (EPC) was procured from Avanti Polar Lipids, Inc. PEGDA (MW: 575Da, 2000Da, 4000Da), DFITC (MW: 150000Da), Iron (III) Chloride Hexahydrate, Ammonium Thiocyanate, Lithium phenyl-2,4,6-trimethylbenzoylphosphinate (LAP), chloroform (ethanol stabilized), Tetramethylrhodamine Isothiocyanate-Dextran (DRITC), Sodium Chloride (NaCl), Potassium Chloride (KCl), Triton™ X-100 were obtained from Sigma–Aldrich (Singapore). PELCO NetMesh™ TEM support Grids are obtained from Ted Pella, Inc. Nuclepore™ Polycarbonate Track-Etched Membranes are obtained from Fisher Scientific Pte Ltd. Spectra/Por^®^ Biotech Cellulose Ester (CE) Membrane (1000 kDa) were procured from Thermo Fisher Scientific. Primary Human Dermal Fibroblasts (HDFs, ATTC^®^ PCS-201-010TM, US) were kindly gifted from Prof. Ng Kee Woei’s research group.

### 2.2 Fabrication of DFITC-loaded PEGDA NLG

PEGDA NLGs were prepared using the thin film hydration method (Zhang, 2017). In brief, EPC was dissolved in chloroform and added into a round bottom flask. The lipid solution was dried using a rotary evaporator at 150 rpm for 1 h, in a water bath at 40°C. The resulting lipid thin film was hydrated at 37°C with solution containing PEGDA hydrogel precursor, DFITC (3 mg/ml) and LAP (0.5 mg/ml), dissolved in phosphate buffered saline (PBS). The thin film was hydrated using a rotary evaporator (without the use of a vacuum pump), at 200 rpm for 1 h. The resulting multilamellar vesicles (MLV) solution was then extruded through polycarbonate membranes of decreasing pore sizes (800 nm, 400 nm, 200 nm) until large unilamellar vesicle (LUVs) of a desired size of between 150 nm and 180 nm are obtained. The LUVs were then purified using ultracentrifugation at 300,000 G and 4°C for 1 h. The resulting pellet was resuspended in fresh PBS. The un-crosslinked NLGs were then exposed to ultraviolet (UV) light of 365 nm (VL-8. L, Vilber, 1-2 mW/cm^2^) for 5 min to form crosslinked NLGs *via* photo-polymerization. Bare liposomes are fabricated the same way, with the lipid thin film hydrated with only PBS.

### 2.3 Characterization of PEGDA NLG nanoparticles

#### 2.3.1 Size and zeta potential

The size, zeta potential and polydispersity index (PDI) of the PEGDA NLG nanoparticles were measured using Malvern Nanosizer 2000. The hydrodynamic size of the samples, together with the PDI, are determined through the measurement of the light intensity fluctuations from the Brownian motion of the particles in suspension, using Dynamic Light Scattering (DLS). Samples are diluted 75x in distilled water before measurement with the Nanosizer. In order to affirm the formation of the nanogel core, crosslinked NLG samples are added with 1% Triton™ X-100 before DLS measurements.

#### 2.3.2 Morphology of NLGs

Cryogenic transmission electron microscopy (TEM) was used to image the bare liposomes, un-crosslinked and crosslinked NLG samples’ morphology with their spherical integrity intact. In cryogenic TEM, the samples were quickly frozen to prevent any structural collapse that may happen in slow drying. 3 µL of sample was pipette onto the TEM copper grids and blotted for 1.5 s, then plunged into liquid nitrogen cooled liquid ethane, using the Cryoplunge^®^ three system (Gatan, Inc. United States). This was done at near 100% humidity to produce amorphous ice around the sample to reduce beam induced damage during imaging, while maintaining native conformation. The frozen sample on the grid is then stored in liquid nitrogen prior to imaging on the Carl Zeiss Libra^®^ 120 Plus electron microscope. The images were taken at 31,500 times magnification at low dose, while maintaining the TEM holder at below −170°C using liquid nitrogen.

#### 2.3.3 Drug encapsulation and release

One of the most important factors to consider when using liposomes for delivery system is the encapsulation efficiency of the vesicles, which is the fraction of total drug used during fabrication that would eventually be encapsulated within the liposomal samples, as shown in Eq (1) below,

$$Encapsulation\;Efficiency\;\left(\%\right)=\frac{Amount\;of\;Drug\;Encapsulated}{Total\;Amount\;of\;Drug\;Used}\times 100$$ (1)

The amount of encapsulated drug can be quantified by breaking the un-crosslinked NLG samples and test for the concentration of DFITC. Liposomal samples were first dissolved in absolute ethanol to dissolve the lipid bilayer. The solution was then diluted 5 times with PBS and tested for fluorescence intensity of DFITC using Tecan Microplate reader (Excitation: 490 nm and Emission: 520 nm). The results were then compared against a calibration curve prepared with known concentrations of DFITC dissolved in the same ratio of PBS and ethanol.

The release of DFITC was monitored over a period of 14 days. Un-crosslinked and crosslinked NLG samples were placed in Spectra/Por^®^ cellulose ester dialysis bag (1000kD, MWCO). To achieve sink condition, the bagged samples were suspended in 40 times the volume of PBS with .05% sodium azide and the release bottles were shaken in a 37°C incubator with the speed of 100 rpm. Each sample was done in triplicates for accurate representation. At each time points (Day 1, 3, 7, 10, 14), 1 ml from the release buffer was extracted to test for amount of DFITC released by testing the fluorescence intensity of the sample against a set of known standards, using Tecan Microplate reader. Fresh PBS with .05% sodium azide was replaced after every time point.

#### 2.3.4 Stewart Assay

Stewart Assay was used to quantify the amount of lipid in the samples. The assay quantifies the phospholipids presence based on the formation of complex between phospholipids and ammonium ferrothiocyanate, after extraction by chloroform (Stewart, 1980). Briefly, un-crosslinked NLG samples were dissolved in chloroform and Stewart reagent was added at a 1:1 ratio. Stewart reagent was prepared beforehand by dissolving ammonium thiocyanate and Iron (III) Chloride hexahydrate in DI water. The mixture was then vortexed and then centrifuged at 500 g to separate the two-phase mixture. The organic phase was extracted and its absorbance at 495 nm was tested using Tecan Microplate reader. The measurements were compared against a calibration graph prepared by measuring increasing concentrations of phospholipids. Lipid loss can be calculated using the following equation,

$$Lipid\;loss=\frac{P_{0}-P_{lipo}\;}{P_{0}}$$ (2)

Where P_0_ is the total amount of phospholipid used and P_lipo_ is the amount of phospholipid in NLG samples, measured using Stewart Assay.

#### 2.3.5 Mesh size calculation

The mesh size, or distance between two crosslinking points, of the nanogel core was calculated using the equations based on Flory-Rehner theory (Peppas et al., 2006),

$$\frac{1}{{\bar{M}}_{c}}=\frac{2}{{\bar{M}}_{n}}-\frac{\left(\frac{\bar{v}}{V_{1}}\right)\left[\ln\left(1-v_{2,s}\right)+v_{2,s}+\chi_{1}v_{2,s}^{2}\right]}{\left(v_{2,s}^{1/3}-\frac{v_{2,s}}{2}\right)}$$ (3)

Where ${\bar{M}}_{c}$ is the molecular weight of the polymer chain between two neighboring crosslinking points, $\bar{v}$ is the specific volume of the polymer, ${\bar{M}}_{n}$ is the molecular weight of the polymer chains, V_1_ is the molar volume of solvent, $\chi_{1}$ is the interaction parameter between the polymer and solvent (.426 for PEG in water) (Truong et al., 2012) and $v_{2,s}$ is the polymer volume fraction in swollen state. $v_{2,s}$ is calculated from the dry mass and wet mass of the NLGs, based on the following relationship (Melekaslan et al., 2004),

$$v_{2,s}={\left[1+\frac{\left(\frac{wet\;mass}{dry\;mass}\right)\rho_{polymer}}{\rho_{solvent}}\right]}^{-1}$$ (4)

Where ρ_polymer_ and ρ_solvent_ are the density of polymer and solvent used for swelling, respectively. Following that, the mesh size (ξ) can be correlated with Flory characteristic ratio (C_n_) (PEG: 4.0), molecular weight of repeating unit (M_r_) and C-C bond length (L) by (Peppas et al., 2006),

$$\xi =v_{2,s}^{-1/3}{\left(\frac{2C_{n}{\bar{M}}_{c}}{M_{r}}\right)}^{1/2}l$$ (5)

ξ provided an insight into the diffusional constraints on release from the nanogel core of NLG system. In this work, the wet and dry masses of the fabricated un-crosslinked NLGs (measured after lyophilization), were measured and used to calculate the mesh sizes.

#### 2.3.6 Fluorescence recovery after photobleaching (FRAP)

In order to monitor the diffusion of particles within the nanogel core in NLGs, FRAP was employed. In brief, a region of interest (ROI) (µm^2^) of fluorescence species within the nanogel core was photobleached by exposure to a high intensity laser for a sufficient amount of time. The fluorescence recovery was observed by capturing still images at fixed intervals.

In order to have micrometer size microlipogels (MLG), gentle hydration was explored for the fabrication of MLGs for FRAP. However, we failed to achieve the MLG based on conventional liposome preparation methods. So we explored the preparation of cell membrane based MLGs by an adapted hypo-osmotic method (Jeewantha and Slivkin, 2018), which was used to harvest the cell membrane ghosts of human dermal fibroblasts and encapsulate PEGDA with DRITC. Briefly, cultured HDFs were collected, washed with PBS and resuspended in .6% NaCl for 30 min at 4°C. The swollen cells were then recovered from the hypotonic cell suspension by centrifugation at 5000 *g* for 5 min. The supernatant was discarded and the recovered swollen cells in the pellet were resuspended in PBS solution containing PEGDA of varying molecular weights, LAP and DRITC, similar to the solution used in fabrication of PEGDA NLG, at 37°C for 60 min. After incubation, equal amount of hypertonic 1.5 M KCl was added to the cell suspension to restore the integrity of the HDF cell membrane, incubated at 37°C for 30 min. The gel precursor loaded HDF cells were then centrifuged and washed with PBS at 120000 g for 15 min at 4°C. Following that, the vesicles are UV crosslinked at 365 nm and the resulting cdMLGs were imaged with confocal microscopy to confirm its formation.

FRAP was then performed on the MLGs using a single-mode laser source of 532 nm (100mW, Coherent Inc. Santa Clara, CA), with a 20 μm diameter circular spot being photobleached. Fluorescence micrographs were then captured at 3 s interval for 8–10 mins to monitor the fluorescence recovery. The obtained data was then normalized and accounted for fading (Kang et al., 2015). The diffusion coefficient were estimated using the Soumpasis Equation as below (Kang et al., 2012),

$$D=0.224\frac{r^{2}}{\tau_{1/2}}$$ (6)

Where D is the diffusion coefficient (μm^2^/s), r being the radius of the bleached spot and τ_1/2_ is the half-life of the recovery, or time taken for the recovery to reached 50% of the mobile fraction.

### 2.4 Statistical analysis

OriginPro 2018 was used to perform analysis of variance (ANOVA) to determine the statistical significance for mobile fractions for all PEGDA MW. A *p*-value of smaller than .05 is considered to be significantly different (*p < .05).

## 3 Results

### 3.1 Particle characterisation

**FIGURE 2:**
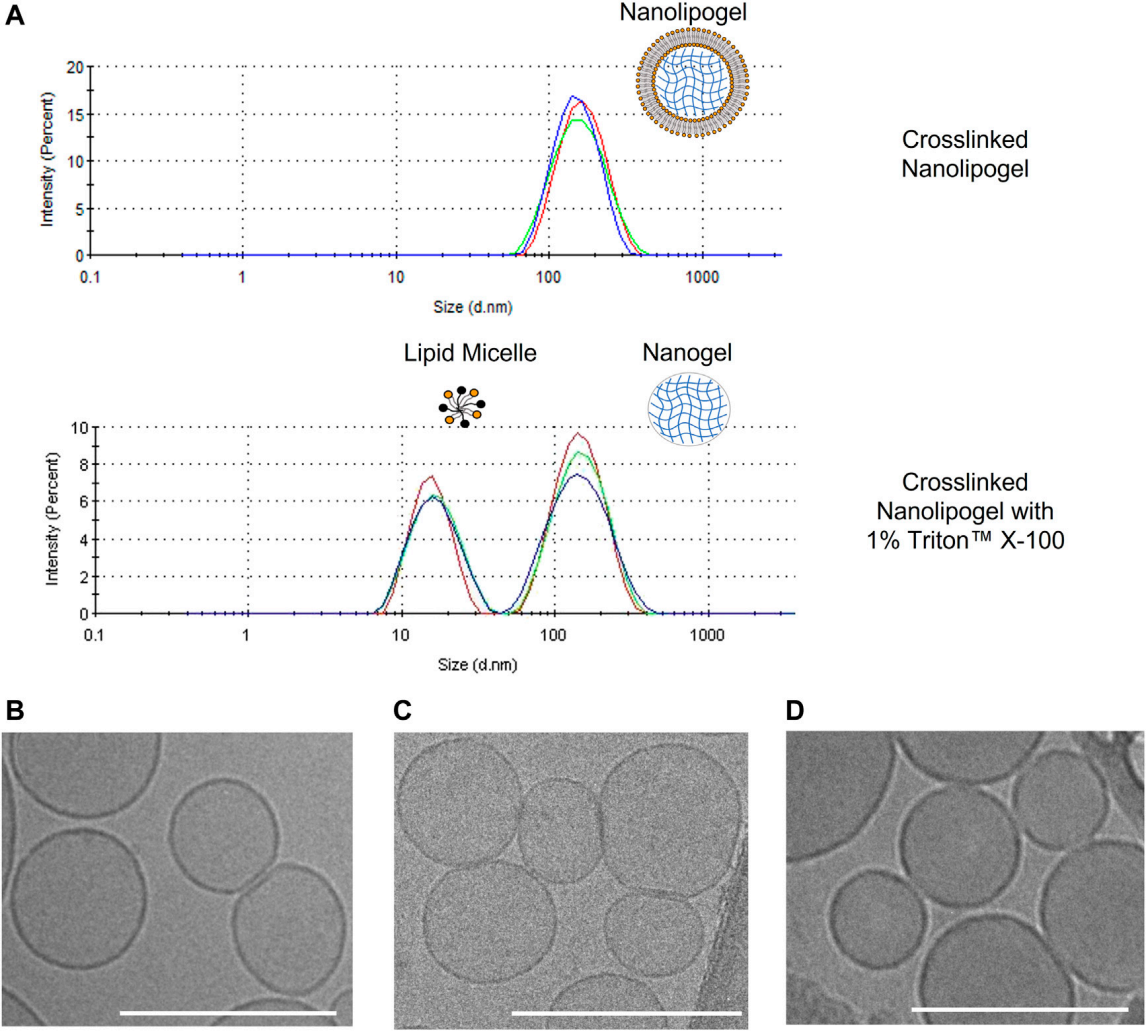
**(A)** DLS size distribution graphs of crosslinked PEGDA NLG and crosslinked PEGDA NLG with 1% Triton™ X-100; Cryogenic-TEM images of **(B)** Bare Liposomes, **(C)** PEGDA 575 uncrosslinked NLGs, **(D)** PEGDA 575 crosslinked NLGs. Unmarked scale bars in the figures represent 200 nm.

As observed in Table 1. all un-crosslinked and crosslinked NLGs showed similar hydrodynamic sizes of about 170 nm and 185 nm respectively, with PDI lower than .15, reflecting homogeneity and narrow size distribution. In Figure 2A, a comparison is shown between the size distribution graph from the DLS measurement of crosslinked NLG and when crosslinked NLGs are added with 1% Triton™ X-100, which showed an additional peak at about 10 nm, apart from the expected nanogel peak around 200 nm. This additional micelle peak is a result of the Triton™ X-100 stripping off the lipid bilayer from the NLGs, leading to the lipids forming micelles in the aqueous environment. Thus, it showed that the NLGs have a structure with a lipid bilayer surrounding a crosslinked nanogel core.

**TABLE 1:** Characterisation of Bare Liposome, uncrosslinked PEGDA NLG and crosslinked PEGDA NLG (n = 3).

PEGDA Mw (Da)		Bare liposome	575	2000	4,000
**Uncrosslinked NLG**	**Size (nm)**	160.5 ± 2.8	165.6 ± 5.4	169.4 ± 2.6	163.8 ± 3.3
	**PDI**	.085 ± .006	.155 ± .007	.109 ± .010	.107 ± .021
**Crosslinked NLG**	**Size (nm)**	–	170.5 ± 5.7	200.3 ± 2.6	188.8 ± 4.2
	**PDI**	–	.117 ± .001	.141 ± .004	.137 ± .089
**Lipid Loss (%)**		10.24 ± 1.22	9.70 ± .40	12.67 ± 1.03	20.29 ± 2.14

Cryogenic TEM is then employed to observe the morphologies of bare liposome, un-crosslinked and crosslinked NLG samples. As seen in Figure 2B, bare liposomes are of nano-spherical shape, consistent with DLS data above. Furthermore, encapsulating PEGDA and DFITC within the core in both un-crosslinked and crosslinked NLGs, in Figures 2C, D respectively, does not cause any significant change in both shape and size.

### 3.2 *In vitro* release studies

**FIGURE 3:**
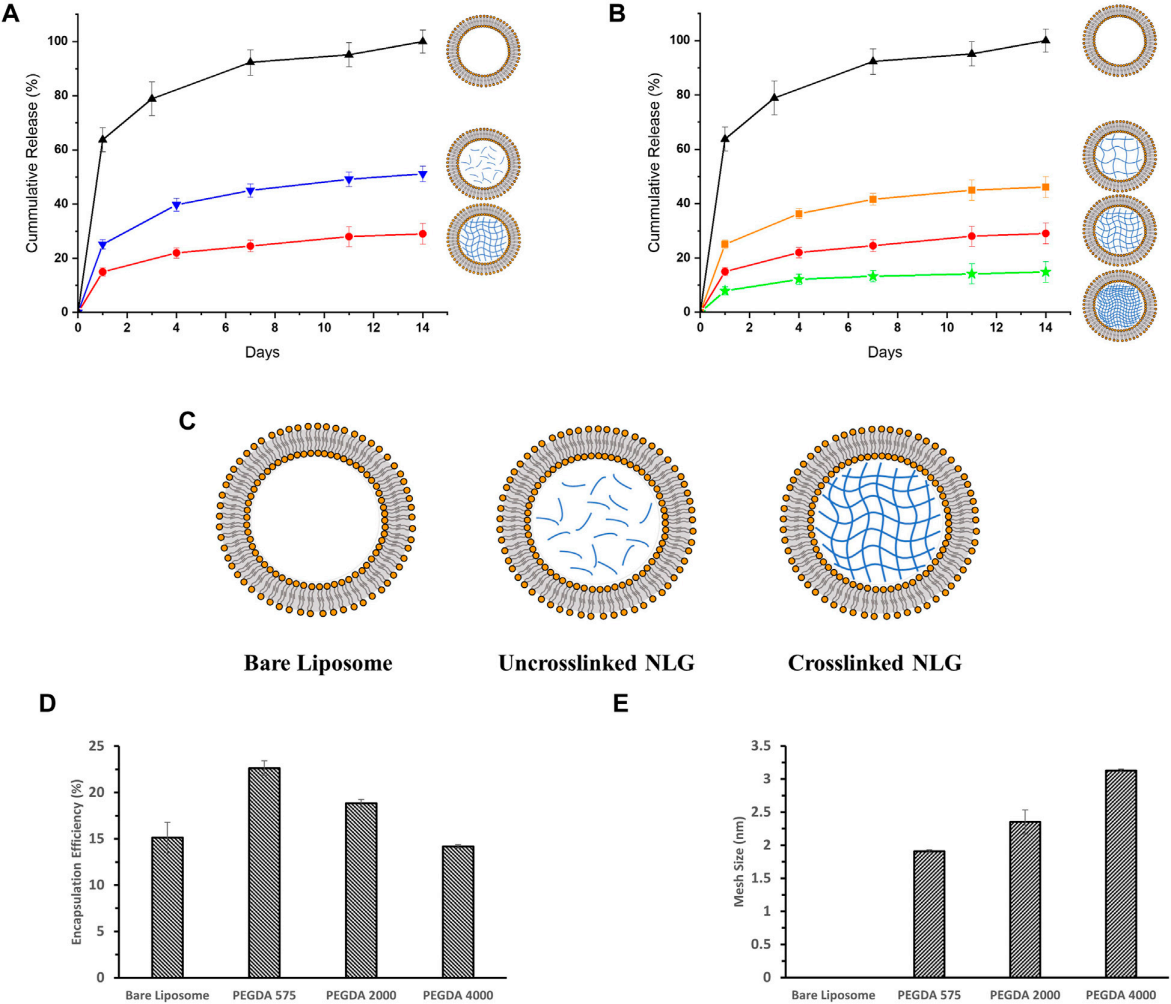
*In-vitro* release profiles for DFITC encapsulated in **(A)** Bare Liposome (Black, Triangle), Uncrosslinked PEGDA 2000 NLG (Blue, Inverted Triangle) and Crosslinked PEGDA 2000 NLG (Red, Circle), **(B)** Bare Liposomes (Black, Triangle), Crosslinked PEGDA 4000 NLG (Orange, Square), Crosslinked PEGDA 2000 NLG (Red, Circle) and Crosslinked PEGDA 575 NLG (Green, Star); **(C)** schematic showing the structural differences between Bare Liposome, Uncrosslinked NLG and Crosslinked NLG; **(D)** encapsulation efficiency and **(E)** mesh size of each PEGDA NLG.

PEGDA of three different molecular weights (575 Da, 2000 Da and 4,000 Da) were used to fabricate NLGs and study the difference in drug release profiles, as shown in Figure 3.

DFITC was encapsulated into PEGDA NLGs to observe the encapsulation efficiency (EE) and release from PEGDA NLGs. As shown in Figure 3D, increasing PEGDA Mw decreases EE and at the same time increases the lipid loss, as shown in Table 1, during fabrication. During which, it was observed that agitation was needed for complete hydration of lipid film for higher PEGDA Mw NLGs, and the pressure needed for extrusion of higher PEGDA Mw NLGs was slightly higher, thus resulting in higher lipid loss and fewer liposome formation. The release of DFITC from PEGDA NLG was then monitored over 14 days at physiological relevant conditions. As shown in Figure 3A, there was burst release of DFITC from bare liposomes, up to 65% on Day 1 and complete release was observed on Day 7. This indicates that the membrane partitioning does not limit the rate of release of DFITC to a huge extent. In comparison, both crosslinked and un-crosslinked NLGs fabricated with PEGDA are able to suppress the initial burst release of DFITC, up to 5-fold and 2-fold more, respectively. Both formulations were also showed to be able to sustain the release over a longer period of time, where the PEGDA NLGs provides better control on the release of DFITC. Furthermore, the Mw of PEGDA, which acts as the macromolecular crowding agent, can be varied to give different mesh sizes and thus different degree of burst release suppression. As the encapsulated molecules are entrapped within the nanogel core in NLG, the mesh size will hinder the diffusion of the molecules through it to varying degrees, as it controls the diffusional path length and steric interactions with the encapsulated molecules (Cao et al., 2020).

It can be seen that un-crosslinked NLGs also show suppression of initial burst release in Figure 3A, down to about 25% in Day 1. This suppression is likely due to the ‘crowding effect’ caused by the presence of PEGDA polymer chains in the core of the liposomes, which disrupts the diffusion of encapsulated DFITC. Therefore, crosslinking of the PEGDA core will further retard the diffusion within the core, resulting in up to 2-fold greater suppression of initial release, as compared to bare liposomes. Next, the effect of crosslinking density in the PEGDA core was examined. According to Figure 3E, PEGDA 575 NLGs shows the smallest mesh size among the three NLGs with different Mw. The smaller mesh size in PEGDA 575 translates to higher crosslinking density (Li and Mooney, 2016), thus allowing it to better entrap encapsulated molecules, such as DFITC in this work. It is noteworthy that when the mesh size are much smaller than the size of DFITC, which is about 9.0 nm (Bu and Russo, 1994; Wen et al., 2013), the mesh size will effectively immobilize the DFITC molecules within the network (Li and Mooney, 2016). Therefore, diffusion of DFITC molecules will then be likely due to reptation only (Peppas et al., 2000). From Figure 3B, we can also see that the suppression of initial burst release is more noteworthy for PEGDA 575 NLG as compared to PEGDA 2000 NLG and PEGDA 4000 NLG, with almost a 10-fold difference from bare liposomes. In addition, the release for DFITC was also slowed to a greater extent of 15% over 14 days for PEGDA 575 NLG, as compared to 20% for PEGDA 2000 NLGs and 40% for PEGDA 4000 NLGs.

Up until now, our results proved that modulation of the release of encapsulated biomolecule can be achieved by varying the mesh size of nanogels; Mw and concentration of PEGDA used in the formation of NLGs have impact on the crosslinking density and therefore mesh size of the NLGs (Cao et al., 2020). The observed trend in cargo release is then validated by performing FRAP on the MLGs, to provide insight on the diffusion across the nanogel core of the NLGs.

### 3.3 FRAP results of MLG

In order to characterize the suppression of burst release in PEGDA NLG and validate the trends in release profile of the PEGDA NLGs, FRAP was performed to understand how the diffusion coefficient of encapsulated molecules varies in different nanogel cores of the NLGs. In FRAP, the irreversible photobleaching is followed by an observable overall recovery of fluorescence, which is due to the diffusion of the neighboring fluorescent species molecules into the photobleached area and the photobleached species molecules out of the area, as a result of the constant ensemble diffusion in the nanogel core (Mika and Poolman, 2011). By observing the recovery of fluorescence, which is a direct result of the constant diffusion of encapsulated fluorophore in the nanogel core, we can correlate that to the diffusion coefficient of the encapsulated fluorophore. However, due to limitations of the size of the photobleaching spot required, micrometer sized MLGs are needed for the study. Fabrication of micrometer sized GUVs commonly involves electroformation (Le Berre et al., 2008) as well as gel-assisted formation (Weinberger et al., 2013). Even though the use of electroformation technique produces GUVs with fewer structural defects and gives high yield (Patil and Jadhav, 2014), it is less efficient for PBS-based solutions due to screening of electric field effect in the solution and electrostatic forces on the phospholipid layers (Li et al., 2016; Lefrançois et al., 2018). Furthermore, purification after GUV formations without leakage of encapsulated solution is also an issue, in order for UV crosslinking of MLGs. Similarly, gel assisted formation of GUVs has some limitations for our work as it involves the swelling of the PVA gel in the PEGDA solutions, after which the phospholipid layer will detach to form GUVs(Weinberger et al., 2013). However, little is known on how the swelling effect will affect the encapsulation of PEGDA and DFITC into the GUV and a certain degree of agitation is necessary to improve the detachment of GUV from PVA surfaces (Weinberger et al., 2013), which presents a risk for leakage of encapsulated materials.

**FIGURE 4:**
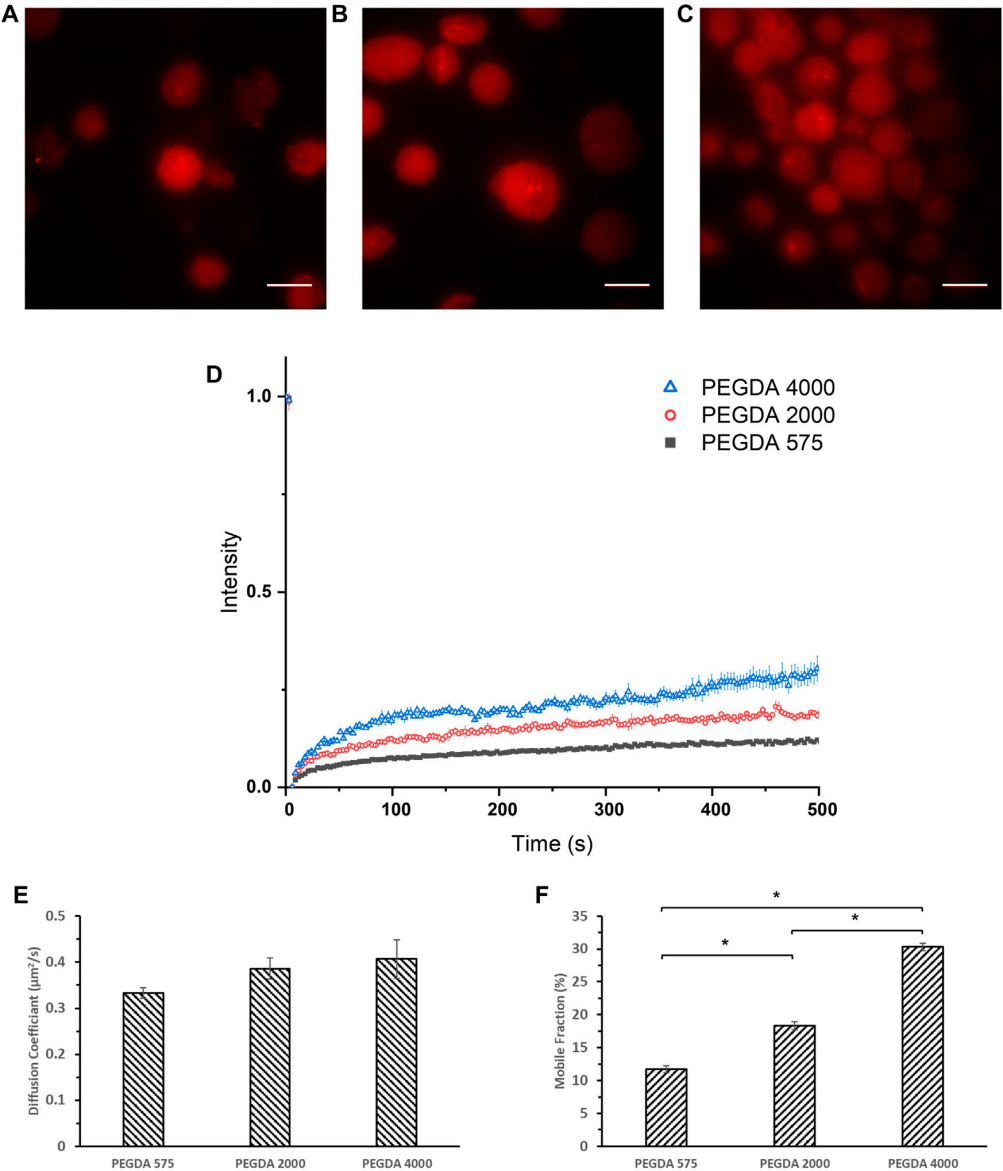
Confocal images of **(A)** crosslinked PEGDA 575 MLG, **(B)** crosslinked PEGDA 2000 MLG, **(C)** crosslinked PEGDA 4000 MLG and **(D)** FRAP recovery curves of crosslinked PEGDA 575 MLG (Black Square), PEGDA 2000 MLG (Red Circle), PEGDA 4000 MLG (Blue Triangle). Unmarked scale bars in the figures represent 20 μm; **(E)** Diffusion Coefficients and **(F)** Mobile Fractions of PEGDA 575, 2000, 4,000 MLGs (*p < .05).

Therefore, we established a new method for MLG formation based on loaded erythrocytes as drug carriers (Jeewantha and Slivkin, 2018), to study the diffusion coefficient of encapsulated molecules using FRAP. Briefly, human dermal fibroblasts were lysed and loaded with PEGDA solutions together with Tetramethylrhodamine Isothiocyanate-Dextran (DRITC), then purified for photo-crosslinking and imaged under a laser confocal microscope. DRITC was used in place of DFITC in order to be bleached by the laser source of the microscope. As seen in Figures 4A–C, vesicles of about 20 μm diameter are consistently fabricated and are loaded with rhodamine tagged dextran. The MLGs were then photobleached with high intensity laser of 532 nm and the intensity recovery was monitored at 3 s intervals. After accounting for fading and background fluorescence, the intensity was plotted as seen in Figure 4D, and the diffusion coefficient, D, for each formulation were estimated and shown in Figure 4E. From Figures 4D, E, it can be seen that the diffusion coefficient of all three samples were comparable of between .333–.407 μm^2^/s. However, what is strikingly different is the mobile fraction, as shown in Figure 4F, which decreases with decreasing Mw of PEGDA. This is in line with the trend in in vitro release studies and mesh size data where the lower PEGDA NLGs shows a larger suppression of the initial burst release. A lower mobile fraction implies that the encapsulated molecules are more tightly trapped within the NLGs and its release is suppressed to a larger extent. The FRAP data thus, lends further support to the foregoing notion that a smaller PEGDA Mw produce NLGs with finer mesh size of the nanogel core, giving a smaller mobile fraction and resulting into greater hinderance to the release of the encapsulated molecular cargo.

It is in order here to note that while a direct positive correlation exists between the mesh size produced by lower molecular weight PEGDA and the mobile fraction, diffusion constants remain unaffected by the mesh size. This then suggests that the effective viscosity and fluidity of the aqueous phase medium of the encapsulated bulk is not affected by the well-hydrated PEGDA gel. We reason that the changes in mobile fraction reflect that a greater proportion of DFITC (i.e., cargo) molecules become immobile because of surface interactions with the finer mesh.

## 4 Discussion

Lipid based nanoparticles, such as liposomes, provides a modifiable alternative for drug delivery systems to improve systemic circulation and biocompatibility. However, it also comes with its own limitations that includes initial burst release of encapsulated hydrophilic molecules and immunogenicity issues. In our work, we introduced PEGDA NLGs with a cell mimicking and tunable design that can be fabricated easily with a one pot method. PEGDA is chosen as the macromolecular crowding agent as it is non-toxic, biocompatible and without harmful degradation by-product (Sabnis et al., 2009; Durst et al., 2011; Browning et al., 2014; Liu et al., 2017; Stillman et al., 2020). In the NLG structure, the nanogel core surrounded by a phospholipid bilayer serves to mimic the natural structure of cells where the cytoplasm is surrounded by the cell membrane, as depicted in Figure 1. In this regard, PEGDA NLG is used as a model system to establish the macromolecular crowding effect and it is being utilized to investigate how the extent of crowding would impact the diffusion coefficient of the drug molecules or biomolecules. PEGDA NLGs can be fabricated with different Mw of PEGDA to encapsulate hydrophilic biomolecules and sustained its release while suppressing its initial burst release. PEGDA solution with DFITC was used to hydrate a thin film of Egg PC, allowing for the self-assembly of lipids into lipid vesicles, encapsulating PEGDA and DFITC. Serial extrusions of the multilamellar vesicles through polycarbonate filter membrane allows for uniformed large unilamellar vesicles (LUV). After purification of the LUVs *via* ultra-centrifugation, the aqueous cores were photo crosslinked to form nanogel cores within the lipid bilayer. This template can be used for different Mw of PEGDA, with homogenous and controlled size being achievable.

**FIGURE 5:**
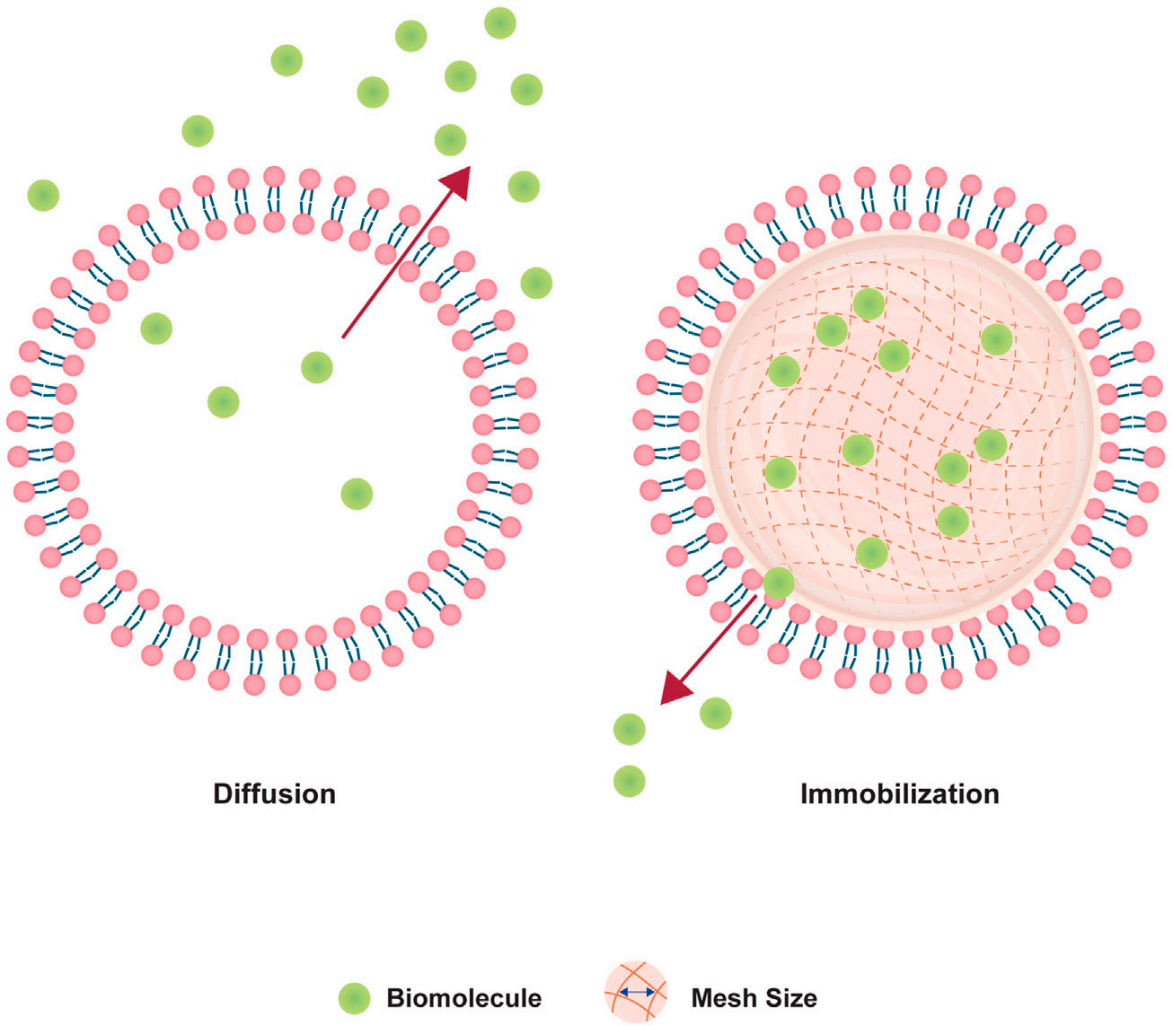
Schematic depicting the effect of crosslinking nanogel core on diffusion of encapsulated biomolecule.

We have also shown that the one pot fabrication method can be used to fabricate homogeneous PEGDA NLG nanoparticles, with PDI of less than .2, for encapsulation of hydrophilic cargos such as DFITC. Further, cryogenic TEM images of the NLG nanoparticles in Figure 2 confirmed the spherical and core shell structures, with sizes that are consistent with DLS data in Table 1. In order to sustain the release of DFITC, DFITC was introduced together with PEGDA during hydration and the aqueous PEGDA core was then photo-crosslinked to obtain a nanogel core, encapsulating the DFITC. Both crosslinked and un-crosslinked nanogel cores hindered the diffusion of DFITC and resulted in a suppression of initial burst release and sustained release. That was shown in Figure 3, where bare liposomes showed burst release of DFITC of about 65% on the first day while PEGDA NLGs have shown to reduce the initial burst release down to 25% for un-crosslinked PEGDA 2000 NLG and 15% for PEGDA 2000 crosslinked NLGs. This trend is also present in PEGDA 575 NLG and PEGDA 4000 NLG, as shown in Supplementary Figure S1, where crosslinked NLGs showed greater suppression of initial burst release and sustained release. In term of the mesh size effect, crosslinked PEGDA NLGs of lower Mw showed greater suppression of initial burst release, as corresponded to mesh size data in Figure 3E, where mesh size is the smallest for PEGDA 575 NLG, which is only 80% of the mesh size of PEGDA 2000 NLG and 60% of PEGDA 4000 NLG’s mesh size. In un-crosslinked NLGs, the presence of PEGDA polymer chains within the core provides a certain degree of steric hindrance on the diffusion by having a crowding effect on the encapsulated biomolecules. Even though the uncrosslinked PEGDA could possibly be diffusing out of the NLG at the same time, it does not result in accelerated release of encapsulated DFITC will still suppress the initial burst release and sustain the release, as shown in the release profiles in Figure 3 and Supplementary Figures S1, S2. In the NLGs where the nanogel cores are crosslinked, the diffusion of the encapsulated biomolecules is hindered by the mesh network of the nanogel by steric hindrance, especially when the mesh size is similar to or smaller than the size of the biomolecule (Li and Mooney, 2016; Cao et al., 2020), as depicted in Figure 5.

As diffusion is key in our study in the release of DFITC from PEGDA NLGs, FRAP was conducted on the particles to understand its diffusion kinetics. Here we introduced a novel method to fabricate micrometer sized PEGDA MLGs, by lysing and loading human dermal fibroblasts. Confocal images in Figure 4 confirmed that MLGs were able to be fabricated with sizes around 20 μm consistently. The MLGs were then photobleached and monitored for fluorescence recovery. From the recovery curves in Figure 4D, the diffusion coefficient and mobile fraction was determined and presented in Figures 4E, F which showed that the mobile fraction decreases as Mw of PEGDA decreases. This correlates with the mesh size data to validate the greater suppression of initial burst release for lower PEGDA Mw NLGs, where the smaller mesh sizes coincide with lower mobile fractions, or spaces where the encapsulated DFITC can diffuse in. Although FRAP was conducted on cell membrane coated MLG as compared to EPC coated NLGs for release studies, the difference between cell membrane and EPC will not significantly affected the diffusion studies performed using FRAP as the ROI monitored for photobleaching and recovery is only selected within the nanogel core. Residual macromolecular substances and organelles are also limited to the membrane layer, and even if there are traces of them within the microgel core, the predominant effect is still the fine mesh size of the crosslinked gel core immobilizing the encapsulated cargo, which are of much bigger scale than any trace interactions with the residual organelles.

Taking the data together, we have shown that PEGDA NLGs can be easily fabricated and are able to encapsulate hydrophilic biomolecules and suppress its initial burst release. PEGDA NLG system also provides a tunable core by using different Mw PEGDA, in which it could be varied and/or crosslinked, to suppress the initial release and sustain its release to different extends. When compared together, as shown in Supplementary Figure S2, it can be seen that the degree of suppression of initial burst release and accumulated release of encapsulated DFITC differs for all formulations, allowing for a wide range of options to select from for the most suitable formulation for a multitude of different applications, where different rates of release are required. For instance, PEGDA 575 NLGs, which only release 15% of the encapsulated DFITC, can be used in immune response applications or in tumour chemotherapy, where the encapsulated biomolecules or even drug, can be kept within the core and be only released after ingestion by inflammatory cells such as resident macrophages (Rooijen and Sanders, 1994). Lysosomal phospholipases in macrophages disrupts the bilayer of the NLG, and the PEGDA nanogel core will undergo phagocytosis and endocytosis (Rooijen and Sanders, 1994; Anselmo et al., 2015), delivering the encapsulated biomolecule. In addition, it can also be observed in Supplementary Figure S2 that the suppression of burst release is similar between uncrosslinked PEGDA 2000 NLG and crosslinked PEGDA 4000 NLG, and between uncrosslinked PEGDA 575 NLG and crosslinked PEGDA 2000 NLG. This opens up the possibility to choose between a crosslinked NLG or a uncrosslinked NLG where a certain release profile is required. In the aforementioned immune response or tumour chemotherapy, we can possibly have a uncrosslinked PEGDA NLG of smaller MW than 575 that may give the same burst release suppression and cumulative release but allows for a much distinct release after the lipid bilayer is ingested by inflammatory cells, as the encapsulated biomolecule are not immobilized by a crosslinked nanogel’s mesh network. On the other hand, for PEGDA 2000 and 4,000 NLGs, the slowed release of 40% may be applied to renal fibrosis treatment by delivering mRNA or cytokines like IL-10 (Nastase et al., 2018), or ocular applications such as delivery to the back of the eye for treating ischemic retinopathies and macular edema (Ozaki et al., 1997; Kompella et al., 2010), where a much slower release over a longer period of time is desired.

Nonetheless, there remains other areas of interest that can be studied, giving us a more complete understanding on the encapsulation, diffusion and release of biomolecules in NLGs, such as the influence from the membrane, if any. EPC was selected in this study for a neutrally charged model membrane system for the introduction of PEGDA nanogel core as it is relatively inert, stable during photopolymerization of NLGs and EPC liposomes has good stability (Yavlovich et al., 2009; Corrales Chahar et al., 2018). Adding of cholesterol, which is present in cell membranes, may be used to further study the membrane properties such as membrane fluidity, permeability or radiation protection (Gaber et al., 2002; Chibowski and Szcześ, 2016). Other phospholipids may also be explored, such as dipalmitoylphosphatidylcholine (DPPC), which will confer temperature sensitivity due to its gel-to-lipid crystalline phase transition at 41°C (Yatvin et al., 1978), or phosphatidylethanolamine (PE) for pH responsiveness (Litzinger and Huang, 1992; Singh et al., 2013). Charged lipids like 1,2-dioleoyl-3-trimethylammoniopropane (DOTAP) and 1,2-distearoyl-sn-glycero-3-phospho- (1′-rac-glycerol) (DSPG) can also be studied for other membrane properties such as increased cellular uptake or skin permeation (Maione-Silva et al., 2019), which may also affect the release of encapsulated biomolecules from such NLGs due to possible interactions with the membrane. These give other avenues to study the membrane effects on encapsulation and release of encapsulated biomolecules.

## 5 Conclusion

In this work, we have successfully characterized the diffusion and release of encapsulated hydrophilic biomolecule from cell mimicking PEGDA NLGs, using a novel method of cell derived microlipogels to conduct FRAP studies. Our findings can be translated into designing a promising system with a tunable core for better control of the release of hydrophilic biomolecules. Through the release studies, we can show that the PEGDA nanogel cores used in the study is able to hinder the diffusion of encapsulated biomolecules and suppress its release, while sustaining its release up to 14 days. Our experiments also proved that crosslinking the NLGs will give even greater suppression than un-crosslinked cores, and thus by changing the Mw of PEGDA, we can control both the burst release suppression as well as the subsequent sustained release. Lower MW PEGDA NLG has a smaller mesh size, due to higher crosslinking density, which will hinder and immobilize the encapsulated hydrophilic biomolecule to a greater extend. We have successfully validated this using FRAP on cell derived MLGs which show a lower mobile fraction for smaller MW PEGDA MLGs. Taken together, our developed method to characterize the nanogel core of PEGDA NLGs shows great promise and potential to guide the design of a delivery system for hydrophilic biomolecules. We expect our approach to be extendable to other forms of NLGs with different nanogel cores, encapsulating different biomolecule cargos as well as different coating using different phospholipids for specific targeting for cells or tissue sites.

## Data Availability

The original contributions presented in the study are included in the article/Supplementary Material, further inquiries can be directed to the corresponding authors.
