# Supplementary material for: Cell-mimicking polyethylene glycol-diacrylate based nanolipogel for encapsulation and delivery of hydrophilic biomolecule
Source: Front Bioeng Biotechnol. 2023 Jan 17;11:1113236. doi: 10.3389/fbioe.2023.1113236 (PMC9888760; doi:10.3389/fbioe.2023.1113236)
Supplement: Supplementary file 1 [file DataSheet1.PDF]

Supplementary Material

1 Supplementary Figures

1.1 In Vitro Release Study

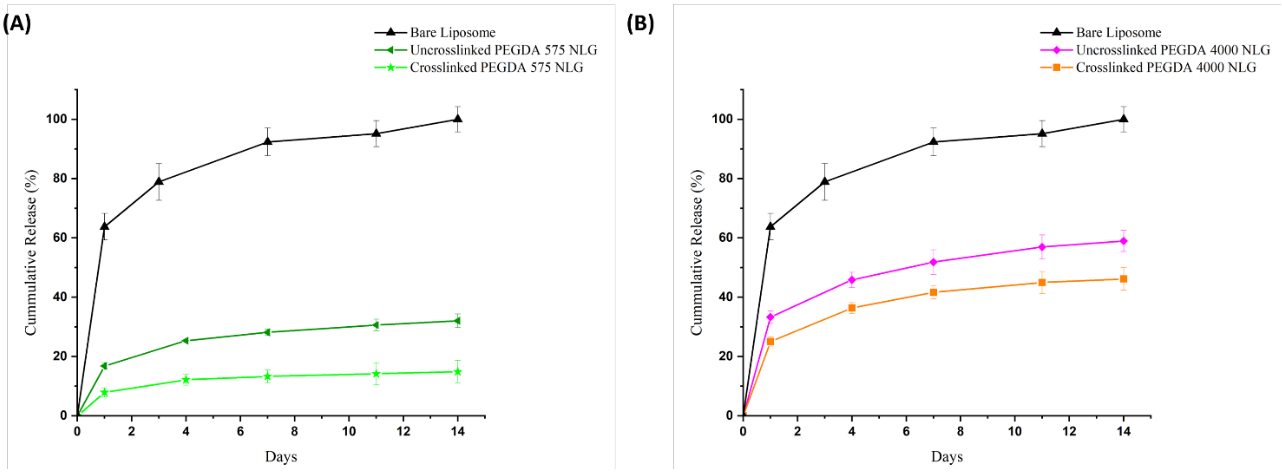

Supplementary Figure 1. *In-vitro* release profiles for DFITC encapsulated in (**A**) Bare Liposome (Black, Triangle), Uncrosslinked PEGDA 575 NLG (Dark Green, Left Pointing Triangle) and Crosslinked PEGDA 575 NLG (Light Green, Star); and (**B**) Bare Liposome (Black, Triangle), Uncrosslinked PEGDA 4000 NLG (Pink, Diamond) and Crosslinked PEGDA 4000 NLG (Orange, Square)

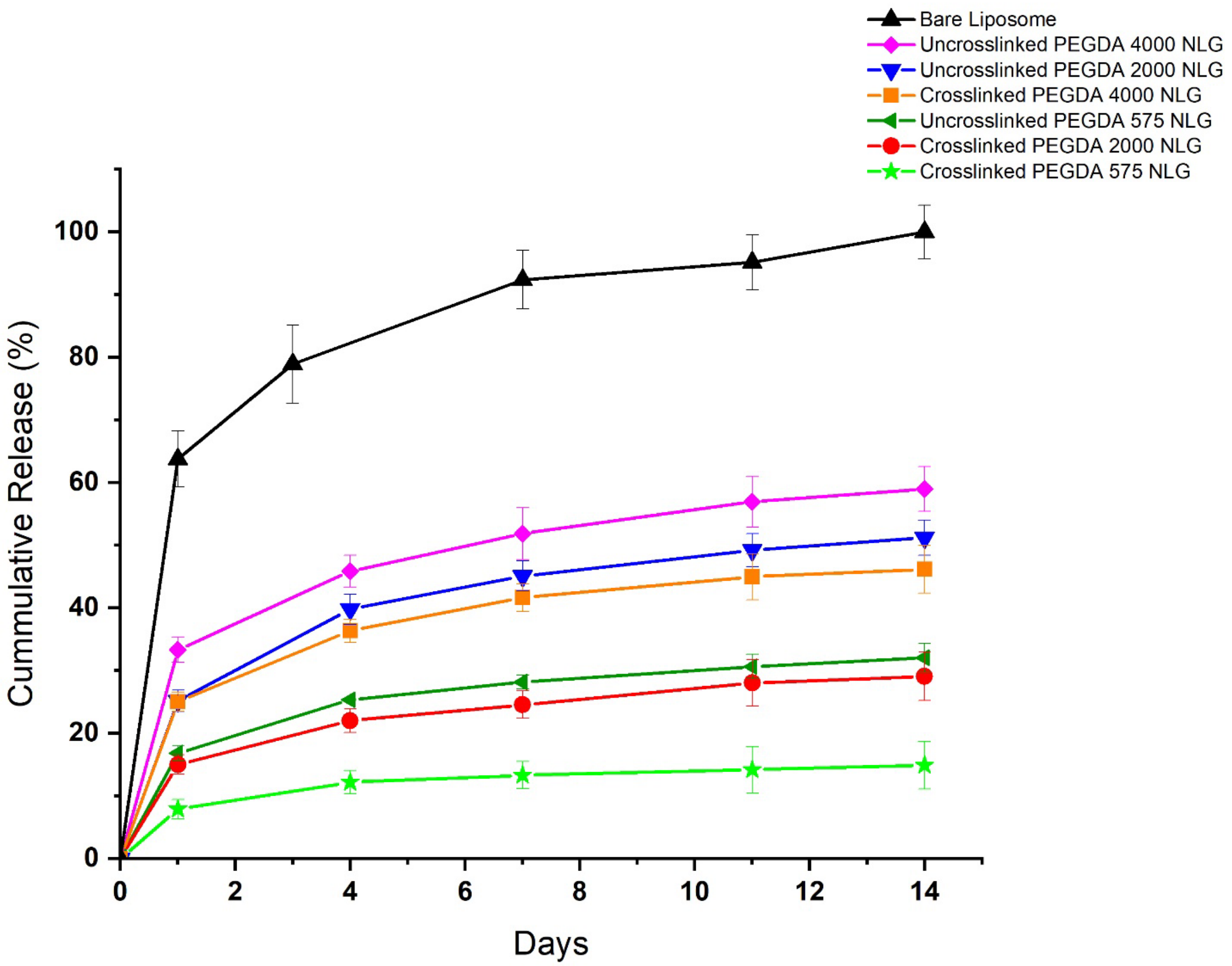

Supplementary Figure 2. *In-vitro* release profiles for DFITC encapsulated in Bare Liposome (Black, Triangle), Uncrosslinked PEGDA 4000 NLG (Pink, Diamond), Uncrosslinked PEGDA 2000 NLG (Blue, Inverted Triangle), Crosslinked PEGDA 4000 NLG (Orange, Square), Uncrosslinked PEGDA 575 NLG (Dark Green, Left Pointing Triangle), Crosslinked PEGDA 2000 NLG (Red, Circle) and Crosslinked PEGDA 575 NLG (Light Green, Star)
